# Effects of *Nigella sativa* and its constituents on total white blood cells count and airway responsiveness value in ovalbumin-induced asthma models: A preclinical systematic review and meta-analysis

**DOI:** 10.22038/ajp.2025.25965

**Published:** 2025

**Authors:** Elmira Mohammadzadeh, Hassan Ghobadi, Jafar Mohammadshahi, Farzin Aslani, Mohammad Hossein Boskabady, Mohammad Reza Aslani

**Affiliations:** 1 *Lung Diseases Research Center, Ardabil University of Medical Sciences, Ardabil, Iran*; 2 *Department of Orthopedics, Imam Khomeini Hospital, Tehran University of Medical Sciences, Tehran, Iran*; 3 *Applied Biomedical Research Center, Mashhad University of Medical Sciences, Mashhad, Iran*

**Keywords:** Asthma, Nigella sativa, Carvacrol, Thymol, Thymoquinone, Alpha-hederin, Ovalbumin, Meta-analysis

## Abstract

**Objective:**

Evidence indicates that *Nigella sativa* (NS) and its key compounds such as carvacrol, thymoquinone, thymol, and α-hederin exhibit properties that reduce inflammation, act as antioxidants, and modulate the immune system. This meta-analysis investigated the preclinical evidence of NS reported in animal models of ovalbumin (OVA)-induced asthma.

**Materials and Methods:**

Studies done on NS and its components in animal models of OVA-induced asthma in all published articles up to July 2024 were searched in Scopus, PubMed, and Web of Science databases. The studies underwent assessment of methodological quality utilizing the 15-point CAMARADES checklist. MedCalc software was utilized for performing the data analysis.

**Results:**

Sixteen studies involving a total of 486 animals, with 243 in the intervention group and 243 in the ovalbumin-induced group were analyzed. In the meta-analysis results, it was shown that NS and its components notably decreased total white blood cells (WBC), eosinophils, lymphocytes, and neutrophils. Additionally, NS caused a shift in the half of the maximum effective concentration (EC50) curve to the right and decreased the maximum response rates and tracheal OVA-response in experimental animals.

**Conclusion:**

NS, and its components, could potentially influence asthma induced by OVA in animals by improving airway responsiveness and exhibiting anti-inflammatory and anti-oxidant properties. Consequently, it is recommended that NS be evaluated in additional clinical trials for patients with asthma.

## Introduction

Asthma, a respiratory disease, is characterized by the presence of hyper-responsive airways as its main feature (Bradding et al. 2024). Furthermore, numerous studies have documented histopathological alterations like smooth muscle hypertrophy, excessive mucus production, fibrosis, and narrowing of the airways in the development of asthma (Huang and Qiu 2022; Keyhanmanesh et al. 2018). The pathophysiology of asthma involves airway inflammation, resulting in immune cell and mediator responses (Aslani et al. 2016a). A variety of immunological components play a role in asthma, such as lymphocytes, mast cells, eosinophils, neutrophils, dendritic cells, macrophages, airway resident cells, and epithelial cells (Aslani et al. 2022a; Qin et al. 2024; Saadat et al. 2020). Furthermore, asthma is influenced by immune factors such as inflammatory mediators, cytokines, chemokines, leukotrienes, and nitric oxide (Hammad and Lambrecht 2021). In asthma, the immune system triggers Th2 cells to release lymphokines like interleukin (IL)-4, IL-15, IL-13, and IL-33 (Ayakannu et al. 2019). Exacerbation of the disease is linked to the activation of both Th1 and Th2 immune responses in treatment-resistant patients and those with severe asthma (Ghobadi et al. 2019; Luo et al. 2022). A new phenotype in asthma pathogenesis has been observed with the activation of Th17 cells in studies involving animals with obesity-related asthma (Akhavanakbari et al. 2019; Aslani et al. 2022c).

The current trend towards using herbal treatments as a substitute option is due to their multiple advantages and limited adverse reactions (Ghasemi et al. 2021; Khazdair et al. 2021; Rahimi et al. 2022). For a considerable period, the black seed plant has served medicinal purposes. Black seed, scientifically identified as *Nigella sativa* (NS), is classified within the Ranunculaceae family (Ahmad et al. 2021). This plant is native to areas such as the Mediterranean Sea, Southwest Asia, Southern Europe, and North Africa, and it is also found in other regions such as India, Pakistan, and various regions of Iran (Ahmad et al. 2021). The ancient Egyptians and Greek physicians turned to the seeds of the black seed plant for treating disorders like nasal congestion, coughs, headaches, asthma, immune system support, allergies, and as a diuretic with the intention of triggering menstruation and increasing milk production (Liao et al. 2021; Saadat et al. 2021). The compounds linked to NS activities remain largely unknown. The chemical composition of NS consists of fixed oil (24.76-40.35%), volatile oil (0.5–1.6%), saponins, alkaloids, and small quantities of other compounds (Koshak et al. 2018). The oils are composed of linoleic, oleic, palmitic, dihomolinoleic, eicosadienoic acids, myristic acids, beta-sitosterol, arachidonic, stearic, cycloeucalenol, cycloartenol, sterol esters and sterol glucosides (Gholamnezhad et al. 2019). A crucial element within NS, thymoquinone (TQ) has been recognized as a pivotal active ingredient in both human and animal research. Carvacrol, thymohydroquinone, dithymoquinone, thymol, and a-hederin are among the other compounds examined in various studies (Hussain et al. 2024). Through pharmacological research, it has been determined that NS and its active components exhibit various properties, including anti-inflammatory capabilities, pressure lowering effects, anti-cancer, smooth muscle relaxant, pain relieving, anti-diabetic, antioxidant, anti-convulsant, anti-ischemic, and diuretic actions (Koshak et al. 2018; Wahab and Alsayari 2023). 

Several comprehensive reviews and meta-analyses have explored the impact of NS in clinical trial investigations (Han and Shi 2021; Kavyani et al. 2023; Mohit et al. 2020; Montazeri et al. 2021). While many of these investigations have proven NS to be effective in clinical trials, they primarily highlight its role in influencing oxidative stress and inflammation markers in various diseases. Furthermore, the meta-analysis of clinical trial studies included only a small number of studies and had a limited sample size (Han and Shi 2021; Sahebkar et al. 2016). Thus, it appears that additional research is required to investigate the efficacy of NS in individuals with asthma. 

Given that the role of NS in animal studies of asthma models has been well studied, the aim of the current study was to investigate its effects on airway inflammation and the severity of airway responsiveness in more detail. Even though animal research has greatly influenced scientific progress, contradictory results continue to complicate matters. Among the plants that have been of interest in ovalbumin-induced asthma models in animal studies are NS and its active ingredients. As a result, the present study conducted a comprehensive review and meta-analysis of the ovalbumin-induced asthma models to identify the anti-inflammatory and smooth muscle relaxing qualities of NS, carvacrol, TQ, thymol, and α-hederin.

## Materials and Methods

The Preferred Reporting Strategies for Systematic Reviews and Meta-Analyses (PRISMA) guidelines served as the foundation for the conducted systematic review and meta-analysis.

### Database and literature search strategies

Through a comprehensive search of all published articles up to July 2024, researchers located experimental studies on the effects of NS and its constituents in animal models of asthma across PubMed, Web of Science, Science Direct, and Scopus. The keywords used are listed in Table 1. Original research published in languages ​​other than English and Persian was excluded from the study. In addition, a comprehensive manual investigation was carried out on the citation lists of potential articles to discover more studies.

**Table 1:** Search strategy in different databases.

**Databases**	**Search strategies**	**Number**
PubMed	(("Nigella sativa" OR (sativa AND Nigella) OR (cumin AND black) OR "kalonji" OR "Kalonjus" OR "black cumin" OR "black cumins" OR (cumins AND black) OR "thymoquinone" OR "dihydrothymoquinone" OR "2-isopropyl-5-methylbenzoquinone" OR "2-methyl-5-isopropyl-p-benzoquinone" OR "TQ" OR "nigellone" OR "4-terpineol" OR "trans-anethole" OR "carvacrol" OR "p-cymene" OR "alpha-pinene" OR "alpha-hederin" OR "kaempferol glucoside" OR "thymol") AND ("ovalbumin" OR "OVA" OR "ova-sensitization" OR "ovalbumin sensitization" OR "ovalbumin sensitized" OR "OVA sensitized" OR "OVA-sensitized" OR "sensitized" OR "OVA-induced asthma") AND ("airway" OR "lung" OR "pulmonary" OR "asthma" OR "asthmatic" OR "Bronchial Asthma" OR (Asthma AND Bronchial) OR "lung remodeling" OR "pulmonary remodeling" OR "airways" OR "trachea" OR "respiratory" OR "allergic airway" OR "allergic asthma" OR "tracheal"))	39
Scopus	(TITLE-ABS-KEY("Nigella sativa" OR (sativa AND Nigella) OR (cumin AND black) OR "kalonji" OR "Kalonjus" OR "black cumin" OR "black cumins" OR (cumins AND black) OR "thymoquinone" OR "dihydrothymoquinone" OR "2-isopropyl-5-methylbenzoquinone" OR "2-methyl-5-isopropyl-p-benzoquinone" OR "TQ" OR "nigellone" OR "4-terpineol" OR "trans-anethole" OR "carvacrol" OR "p-cymene" OR "alpha-pinene" OR "alpha-hederin" OR "kaempferol glucoside" OR "thymol") AND TITLE-ABS-KEY("ovalbumin" OR "OVA" OR "ova-sensitization" OR "ovalbumin sensitization" OR "ova sensitized" OR "ova-sensitized" OR "sensitized" OR "OVA-induced asthma") AND TITLE-ABS-KEY("airway" OR "lung" OR "pulmonary" OR "asthma" OR "asthmatic" OR "Bronchial Asthma" OR (Asthma AND Bronchial) OR "lung remodeling" OR "pulmonary remodeling" OR "airways" OR "trachea" OR "respiratory" OR "allergic airway" OR "allergic asthma" OR "tracheal"))	49
Web of sciences	((TS=("Nigella sativa" OR (sativa AND Nigella) OR (cumin AND black) OR "kalonji" OR "Kalonjus" OR "black cumin" OR "black cumins" OR (cumins AND black) OR "thymoquinone" OR "dihydrothymoquinone" OR "2-isopropyl-5-methylbenzoquinone" OR "2-methyl-5-isopropyl-p-benzoquinone" OR "TQ" OR "nigellone" OR "4-terpineol" OR "trans-anethole" OR "carvacrol" OR "p-cymene" OR "alpha-pinene" OR "alpha-hederin" OR "kaempferol glucoside" OR "thymol") AND TS=("ovalbumin" OR "OVA" OR "ova-sensitization" OR "ovalbumin sensitization" OR "ova sensitized" OR "ova-sensitized" OR "sensitized" OR "OVA-induced asthma") AND TS=("airway" OR "lung" OR "pulmonary" OR "asthma" OR "asthmatic" OR "Bronchial Asthma" OR (Asthma AND Bronchial) OR "lung remodeling" OR "pulmonary remodeling" OR "airways" OR "trachea" OR "respiratory" OR "allergic airway" OR "allergic asthma" OR "tracheal"))	50

### Eligibility criteria

The meta-analysis included animal studies that investigated the effects of NS and its active components on the intervention group ovalbumin (OVA) and intervention) in comparison to the control group with OVA-induced asthma. 

The set measures to exclude bias were: 1- Sensitization caused by intraperitoneal injection and nebulizer challenge with OVA; 2- The subjects in the experimental cohort were treated with either NS or its fundamental ingredients in a single therapy for each dosage given. Animals in the control group were administered non-functional fluid, specifically normal saline, with no further treatments provided; 3- Investigating the effect of NS or its active constituents on WBC count and different cell types such as eosinophil, monocyte, neutrophils, and lymphocyte is crucial for this study’s primary outcome. A secondary goal of the case evaluation was to explore how NS and its active constituents affected tracheal responsiveness following OVA-sensitization.

Treatments involving alternative NS compounds or their active ingredients, sensitization models not incorporating OVA, studies without control groups, and those with inadequate data were disregarded.

### Data extraction

Two authors (E.M and F.A.) extracted the following items independently from the studies that were included: 1- the asthma induction model utilized, alongside the year of publication and primary author identified.; 2- The characteristics of the examined animals are marked by a range of numbers, species types, and sexes; 3- Details regarding the intervention group were provided, which included the specifics of the intervention utilized (*Nigella sativa* extract, TQ, carvacrol, thymol, or α-hederin), dosage of the intervention, duration of the intervention, method of administration, and details on the control group; 4- average and standard deviation for each data. When the information did not match up, another author (MR.A.) got involved. The investigation carefully considered each dosage of intervention, acknowledging the diverse effects that can result from different levels of dosing. If meta-analysis data is not available or results are only shown graphically, authors were contacted at least three times. If a response is not given, the digital ruler software measures and removes the data from the graphic charts if no information is present.

### Quality assessment

The Collaborative Evidence-Based Complementary and Alternative Medicine method (CAMARADES) was used to evaluate the quality of the study’s methodology by analyzing animal data in experimental studies. Fifteen different criteria were used to evaluate the quality of research, ranked based on specific indicators from low to high.

### Risk of bias assessment

The risk of bias tool for animal studies developed by SYRCLE was applied to assess potential biases. The adapted tool evaluated twelve inquiries and categorized each research with a bias risk rating of high, medium, or low.

### Statistical analysis

The findings were evaluated using the MedCalc software. The combined effect size for continuous data was estimated using a random effects model, taking into account both the confidence interval (CI) and either the mean difference (MD) or standard mean difference (SMD). Because of the heterogeneity in the studies, results are displayed using the random effect model. The Q-test and I^2^ index were used in evaluating heterogeneity, with significance being established as p<0.10. (I^2^ < 25%, no heterogeneity; I^2^ between 25% and 50%, moderate heterogeneity; I^2^ between 50% and 75%, large heterogeneity; and I^2^ > 75%, extreme heterogeneity). The examination of publication bias involved analyzing funnel plots and conducting Eggers regression tests.

## Results

### Study Selection

**Figure 1:**
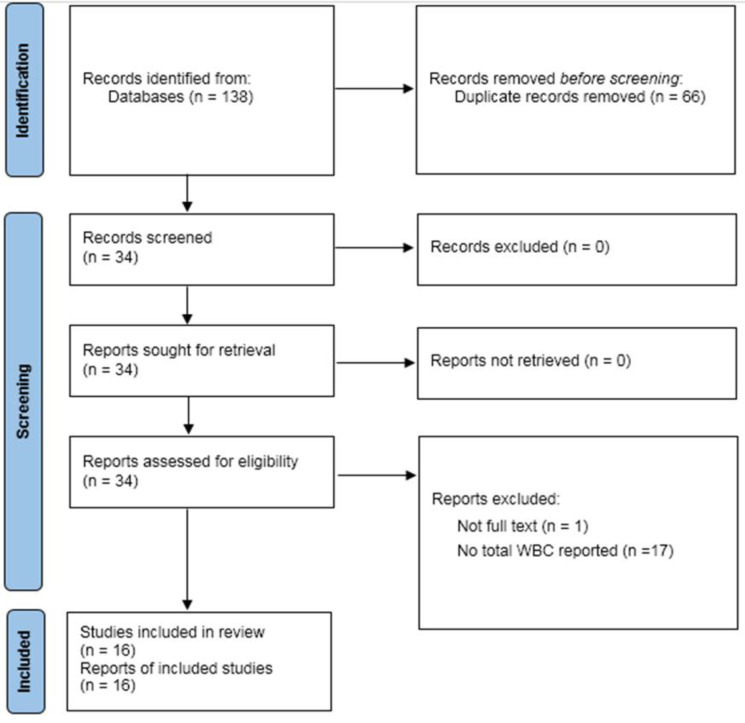
Summary of the process of identifying candidate studies.

The electronic database search yielded a total of 138 records out of which 72 were singled out as distinct. Following a thorough examination of their titles and abstracts, it was discovered that 38 articles did not meet the necessary criteria; therefore, they were excluded from further consideration, allowing for a focused review of the remaining 34 articles in line with the inclusion criteria. A total of seventeen reports were dismissed because they did not contain sufficient information regarding white blood cell (WBC) counts and differential cell analysis. The full text of the article was not available (Abbas et al. 2005). A total of sixteen articles passed the evaluation process and qualified for inclusion in the meta-analysis with their full texts closely examined (Ammar el et al. 2011; Balaha et al. 2012; Boskabady and Jalali 2013; Boskabady et al. 2011; Boskabady et al. 2014; El Gazzar et al. 2006a; El Gazzar et al. 2006b; El Mezayen et al. 2006; Keyhanmanesh et al. 2010; Keyhanmanesh et al. 2014; Khaldi et al. 2018; Pejman et al. 2014; Rafique et al. 2018; Saadat et al. 2015; Shahzad et al. 2009; Zhou et al. 2014) (Figure 1).

### Study quality

All selected research articles scored above nine in the quality assessment tool consisting of 15 criteria. Every article had underwent peer-review before publication. Additionally, every examination provided information on the testing procedures, dosage of intervention, and specific method of intervention used (NS extract, TQ, carvacrol, thymol or α-hederin). Despite this, conflict of interest was observed in 6 separate studies (Keyhanmanesh et al. 2014; Khaldi et al. 2018; Rafique et al. 2018; Saadat et al. 2015; Shahzad et al. 2009; Zhou et al. 2014). In contrast, there are no existing studies on the procedures for calculating sample size, conducting blinded model induction, and blinding outcome assessments (Table 2). 

### Risk of bias

Analysis using the SYRCLE risk of bias tool indicated that most of the animal studies showed low risk, with only one study presenting a medium risk (Table 3). 

### Study characteristics

The characteristics of the included studies are summarized in Table 4. In 16 studies, there were 486 animals in total, with the OVA-induced asthma control group and intervention group each containing 243 animals. The research articles included in the study all had publication dates from 2006 to 2018 and were written in English. Five studies utilized BALB/C mice in their experiments (Balaha et al. 2012; El Gazzar et al. 2006a; El Gazzar et al. 2006b; El Mezayen et al. 2006; Zhou et al. 2014), a single study used albino mice (Ammar el et al. 2011), seven studies used guinea pigs (Boskabady and Jalali 2013; Boskabady et al. 2011; Boskabady et al. 2014; Keyhanmanesh et al. 2010; Keyhanmanesh et al. 2014; Pejman et al. 2014; Rafique et al. 2018; Saadat et al. 2015), and two studies used rats (Khaldi et al. 2018; Shahzad et al. 2009). Research was based on males in six studies (Ammar el et al. 2011; Balaha et al. 2012; Keyhanmanesh et al. 2014; Khaldi et al. 2018; Pejman et al. 2014; Saadat et al. 2015), females in two studies (Shahzad et al. 2009; Zhou et al. 2014), and both genders in eight studies (Boskabady and Jalali 2013; Boskabady et al. 2011; Boskabady et al. 2014; El Gazzar et al. 2006a; El Gazzar et al. 2006b; El Mezayen et al. 2006; Keyhanmanesh et al. 2010; Rafique et al. 2018). Asthma models in animals were created using OVA injection and challenge in all experiments. TQ was utilized in seven studies (Ammar el et al. 2011; El Gazzar et al. 2006a; El Gazzar et al. 2006b; El Mezayen et al. 2006; Keyhanmanesh et al. 2010; Keyhanmanesh et al. 2014; Saadat et al. 2015), carvacrol in two studies (Boskabady and Jalali 2013; Boskabady et al. 2014), and thymol in one study (Zhou et al. 2014). Moreover, only NS extract was utilized in five of the studies (Balaha et al. 2012; Boskabady et al. 2011; Khaldi et al. 2018; Rafique et al. 2018; Shahzad et al. 2009), while one study included both TQ and α-hederin individually (Saadat et al. 2015). The intervention values ​​used for TQ were 3 mg/kg (intraperitoneally (I.P.)), 20 μM through Drinking water (orally), 40 μM through Drinking water (orally), and 15 mg/kg/day (orally); the intervention values used for thymol 4 mg/kg (I.P.), 8 mg/kg (I.P.), and 16 mg/kg (I.P.); the intervention values used for α-hederin 0.3 mg/kg (I.P.), and 3 mg/kg (I.P.); used for carvacrol 40 µg/ml (orally), 80 µg/ml (orally), and 160 µg/ml (orally),; used for *NS* oil 4 ml/kg (I.P.); the intervention values used for *NS* extract 0.125 mg/ml (orally), 0.25 mg/ml (orally), 1 ml/kg/day (orally), 4 ml/kg/day (orally), and 5 mg/kg (orally).

**Table 2:** Quality assessment of the included studies.

**S/N**	**Study and year of publication**	**Q1**	**Q2**	**Q3**	**Q4**	**Q5**	**Q6**	**Q7**	**Q8**	**Q9**	**Q10**	**Q11**	**Q12**	**Q13**	**Q14**	**Q15**	**Total score/15**
1	El Gazzar (2006) a	Yes	-	-	-	-	Yes	Yes	Yes	Yes	Yes	Yes	-	Yes	Yes	-	9
2	El Gazzar (2006) b	Yes	-	-	-	-	Yes	Yes	Yes	Yes	Yes	Yes	-	Yes	Yes	-	9
3	El Mezayen (2006)	Yes	-	-	-	-	Yes	Yes	Yes	Yes	Yes	Yes	-	Yes	Yes	-	9
4	Shahzad (2009)	Yes	-	-	-	-	Yes	Yes	Yes	Yes	Yes	Yes	-	Yes	Yes	Yes	10
5	Keyhanmanesh (2010)	Yes	Yes	-	-	-	Yes	Yes	Yes	Yes	Yes	Yes	-	Yes	Yes	-	10
6	Ammar (2011)	Yes	Yes	-	-	-	Yes	Yes	Yes	Yes	Yes	Yes	-	Yes	Yes	-	10
7	Boskabady (2011)	Yes	Yes	-	-	-	Yes	Yes	Yes	Yes	Yes	Yes	-	Yes	Yes	-	10
8	Balaha (2012)	Yes	Yes	-	-	-	Yes	Yes	Yes	Yes	Yes	Yes	-	Yes	Yes	-	10
9	Boskabady, (2013)	Yes	Yes	-	-	-	Yes	Yes	Yes	Yes	Yes	Yes	-	Yes	Yes	-	10
10	Boskabady, (2014)	Yes	Yes	-	-	-	Yes	Yes	Yes	Yes	Yes	Yes	-	Yes	Yes	-	
11	Keyhanmanesh (2014)	Yes	Yes	Yes	-	-	Yes	Yes	Yes	Yes	Yes	Yes	-	Yes	Yes	Yes	12
12	Pejman (2014)	Yes	Yes	Yes	-	-	Yes	Yes	Yes	Yes	Yes	Yes	-	Yes	Yes	-	11
13	Zhou (2014)	Yes	Yes	-	-	-	Yes	Yes	Yes	Yes	Yes	Yes	-	Yes	Yes	Yes	11
14	Saadat (2015)	Yes	Yes	Yes	-	-	Yes	Yes	Yes	Yes	Yes	Yes	-	Yes	Yes	Yes	12
15	Khaldi (2018)	Yes	Yes	-	-	-	Yes	Yes	Yes	Yes	Yes	Yes	-	Yes	Yes	Yes	11
16	Rafique (2018)	Yes	Yes				Yes	Yes	Yes	Yes	Yes	Yes		Yes	Yes	Yes	11

Modified Tool: (Q1) publication in a peer-reviewed journal; (Q2) statement of temperature control; (Q3) random allocation to groups (Q4) allocation concealment (Q5) blinded assessment of outcome; (Q6) description of the test methods (Q7) Description/origin of the intervention/venom (Q8) reports on the dose/concentration of venom and intervention (Q9) appropriate animal/test model (Q10) appropriate control group/test as part of the method (Q11) report of exposure period (Q12) sample size calculation; (Q13) reports of statistical methods employed (Q14) compliance with animal welfare regulations; (Q15) statement of potential conflict of interests.

**Table 3:** Risk of bias in the included studies.

**S/N**	**Study and year of publication**	**Query 1**	**Query 2**	**Query 3**	**Query 4**	**Query 5**	**Query 6**	**Query 7**	**Query 8**	**Query 9**	**Query 10**	**Query 11**	**Query 12**	**RISK**
1	El Gazzar (2006) a	Yes	Yes	Yes	-	Yes	-	-	-	Yes	Yes	Yes	Yes	low
2	El Gazzar (2006) b	Yes	Yes	Yes	-	Yes	-	-	-	Yes	Yes	Yes	Yes	low
3	El Mezayen (2006)	Yes	Yes	Yes	-	Yes	-	-	-	Yes	Yes	Yes	Yes	low
4	Shahzad (2009)	Yes	Yes	Yes	-	Yes	-	-	-	Yes	Yes	Yes	Yes	low
5	Keyhanmanesh (2010)	Yes	Yes	Yes	-	Yes	-	-	-	Yes	Yes	Yes	Yes	low
6	Ammar (2011)	Yes	Yes	Yes	-	Yes	-	-	-	Yes	Yes	Yes	Yes	low
7	Boskabady (2011)	Yes	Yes	Yes	-	Yes	-	-	-	Yes	Yes	Yes	Yes	low
8	Balaha (2012)	Yes	Yes	Yes	-	Yes	-	-	-	Yes	Yes	Yes	Yes	low
9	Boskabady, (2013)	Yes	Yes	Yes	-	Yes	-	-	-	Yes	Yes	Yes	Yes	low
10	Boskabady, (2014)	Yes	Yes	Yes	-	Yes	-	-	-	Yes	Yes	Yes	Yes	low
11	Keyhanmanesh (2014)	Yes	Yes	Yes	Yes	Yes	-	-	-	Yes	Yes	Yes	Yes	low
12	Pejman (2014)	Yes	Yes	Yes	Yes	Yes	-	-	-	Yes	Yes	Yes	Yes	low
13	Zhou (2014)	Yes	Yes	Yes	-	Yes	-	-	-	Yes	Yes	Yes	Yes	low
14	Saadat (2015)	Yes	Yes	Yes	Yes	Yes	-	-	-	Yes	Yes	Yes	Yes	low
15	Khaldi (2018)	Yes	es	Yes	-	Yes	-	-	-	Yes	Yes	Yes	Yes	low
16	Rafique (2018)	Yes	Yes	Yes		Yes	-	-	-	Yes	Yes	Yes	Yes	low

Query 1: Was the allocation sequence adequately generated and applied? Query 2: Were the groups/test sample similar at baseline or homogenous? Query 3: Was the allocation adequately concealed or homogenous test samples? Query 4: Were the animals randomly housed during the experiment? Query 5: Were the test specimen appropriately and uniformly stored during the experiment? Query 6: Were the investigators blinded from knowing which intervention each animal/test group received during the experiment? Query 7: Were animals selected at random for outcome assessment? Query 8: Was the outcome assessor-blinded? Query 9: Were all major outcomes reported? Query 10: Are reports of the study free of selective outcome reporting? Query 11: Was the study apparently free of other problems that could result in a high risk of bias? Query 12: Was the administered dose uniform or the concentration level of the intervention homogenous?

The total WBC and lymphocyte counts were documented in sixteen studies (Ammar el et al. 2011; Balaha et al. 2012; Boskabady and Jalali 2013; Boskabady et al. 2011; Boskabady et al. 2014; El Gazzar et al. 2006a; El Gazzar et al. 2006b; El Mezayen et al. 2006; Keyhanmanesh et al. 2010; Keyhanmanesh et al. 2014; Khaldi et al. 2018; Pejman et al. 2014; Rafique et al. 2018; Saadat et al. 2015; Shahzad et al. 2009; Zhou et al. 2014), while eosinophil levels were included in fifteen studies (Ammar el et al. 2011; Balaha et al. 2012; Boskabady and Jalali 2013; Boskabady et al. 2011; Boskabady et al. 2014; El Gazzar et al. 2006a; El Gazzar et al. 2006b; El Mezayen et al. 2006; Keyhanmanesh et al. 2010; Keyhanmanesh et al. 2014; Pejman et al. 2014; Rafique et al. 2018; Saadat et al. 2015; Shahzad et al. 2009; Zhou et al. 2014), neutrophils in twelve studies (Boskabady and Jalali 2013; Boskabady et al. 2011; Boskabady et al. 2014; El Gazzar et al. 2006a; El Gazzar et al. 2006b; El Mezayen et al. 2006; Keyhanmanesh et al. 2010; Keyhanmanesh et al. 2014; Pejman et al. 2014; Rafique et al. 2018; Saadat et al. 2015; Zhou et al. 2014), and monocytes and macrophages in sixteen studies (Ammar el et al. 2011; Balaha et al. 2012; Boskabady and Jalali 2013; Boskabady et al. 2011; Boskabady et al. 2014; El Gazzar et al. 2006a; El Gazzar et al. 2006b; El Mezayen et al. 2006; Keyhanmanesh et al. 2010; Keyhanmanesh et al. 2014; Khaldi et al. 2018; Pejman et al. 2014; Rafique et al. 2018; Saadat et al. 2015; Shahzad et al. 2009; Zhou et al. 2014). In one study, total WBC and differential cells were counted using serum samples (Boskabady and Jalali 2013) while BALF samples were used in fourteen other studies (Ammar el et al. 2011; Balaha et al. 2012; Boskabady et al. 2011; Boskabady et al. 2014; El Gazzar et al. 2006a; El Gazzar et al. 2006b; El Mezayen et al. 2006; Keyhanmanesh et al. 2010; Keyhanmanesh et al. 2014; Khaldi et al. 2018; Pejman et al. 2014; Saadat et al. 2015; Shahzad et al. 2009; Zhou et al. 2014). Additionally, in another study, BALF and serum samples were utilized independently (Rafique et al. 2018).

Half of the maximum effective concentration (EC_50_) was recorded in six studies (Boskabady and Jalali 2013; Boskabady et al. 2011; Keyhanmanesh et al. 2010; Keyhanmanesh et al. 2014; Pejman et al. 2014; Saadat et al. 2015), contractility was analyzed in six studies (Boskabady and Jalali 2013; Boskabady et al. 2011; Keyhanmanesh et al. 2010; Keyhanmanesh et al. 2014; Pejman et al. 2014; Saadat et al. 2015), and OVA-response was documented in six different studies (Boskabady and Jalali 2013; Boskabady et al. 2011; Keyhanmanesh et al. 2010; Keyhanmanesh et al. 2014; Pejman et al. 2014; Saadat et al. 2015)

**Table 4:** Main characteristics of the studies included in this meta-analysis

**S/N**	**Study (year)**	**Species (sex, n= OVA group /intervention group)**	**N. sativa or ingredients**	**dose and route of administration**	**Duration of study**	**Factors for analysis** **(type of sample)**	**Intergroup differences (OVA vs. intervention)**
1	El Gazzar (2006) a	BALB/c mice (both sexes, 6/6)	TQ	3 mg/kg, IP	30 days	WBC (BALF) Macrophage (BALF) Neutrophil (BALF) Lymphocyte (BALF) Eosinophil(BALF)	<0.05 NS NS NS <0.05
2	El Gazzar (2006) b	BALB/c mice (both sexes, 6/6)	TQ	3 mg/kg, IP	30 days	WBC (BALF) Macrophage (BALF) Neutrophil (BALF) Lymphocyte (BALF) Eosinophil(BALF)	<0.05 NS NS NS <0.05
3	El Mezayen (2006)	BALB/c mice (both sexes, 6/6)	TQ	3 mg/kg, IP	30 days	WBC (BALF) Macrophage (BALF) Neutrophil (BALF) Lymphocyte (BALF) Eosinophil(BALF)	<0.05 NS NS NS <0.05
4	Shahzad (2009)	Rats (female, 10/10)	*N. sativa*	4 ml/kg, IP	22 days	WBC (BALF) Eosinophil (BALF) Macrophage (BALF) Lymphocyte (BALF)	<0.001 <0.01 <0.05 <0.01
5	Keyhanmanesh (2010) a	Guinea pig (both sexes, 7/7)	TQ	20 μM Drink, orally	32	Total WBC (BALF) Eosinophil (BALF) Neutrophil (BALF) Monocyte (BALF) Lymphocyte (BALF) EC50 OVA-response Contractility	<0.001 <0.001 NS <0.001 <0.05 <0.05 <0.001 NS
	Keyhanmanesh (2010) b	Guinea pig (both sexes, 7/7)	TQ	40 μM Drink, orally	32	Total WBC (BALF) Eosinophil (BALF) Neutrophil (BALF) Monocyte (BALF) Lymphocyte (BALF) EC50 OVA-response Contractility	<0.001 <0.001 <0.05 <0.001 <0.05 <0.001 <0.001 <0.05
6	Ammar (2011)	Albino Mice, (male, 10/10)	TQ	15 mg /kg / day, Orally	38 days	WBC (BALF) Lymphocyte (BALF) Monocyte (BALF) Eosinophil (BALF)	<0.001 <0.001 <0.001 <0.001
7	Boskabady (2011) a	Guinea pig (both sexes, 8/8)	*N. sativa*	0.125 mg/ml drink	36 days	WBC (BALF) Eosinophil (BALF) Neutrophil (BALF) Lymphocyte (BALF) Monocyte (BALF) EC50 OVA-response Contractility	<0.01 NS <0.05 NS <0.05 <0.05 <0.05 NS
	Boskabady (2011) b	Guinea pig (both sexes, 8/8)	*N. sativa*	0.25 mg/ml drink	36 days	WBC (BALF) Eosinophil (BALF) Neutrophil (BALF) Lymphocyte (BALF) Monocyte (BALF) EC50 OVA-response Contractility	<0.01 NS <0.05 NS <0.01 <0.01 <0.01 <0.05
8	Balaha (2012) a	BALB/c mice, (male,6/6)	*N. sativa*	1 ml/kg/day, orally	31 days	WBC (BALF) Macrophage (BALF) Eosinophil (BALF) Lymphocyte (BALF)	NS NS NS NS
	Balaha (2012) b	BALB/c mice, (male,6/6)	*N. sativa*	4 ml/kg/day, orally	31 days	WBC (BALF) Macrophage (BALF) Eosinophil (BALF) Lymphocyte (BALF	<0.01 <0.01 <0.01 NS
9	Boskabady (2013) a	Guinea pig (both sexes, 6/6)	Carvacrol	40 µg/ml, orally	35 days	WBC (blood) Eosinophil (blood) Neutrophil (blood) Lymphocyte (blood) Monocyte (blood) EC50 OVA-response Contractility	NS NS NS NS NS <0.001 <0.05 <0.01
	Boskabady (2013) b	Guinea pig (both sexes, 6/6)	Carvacrol	80 µg/ml, orally	35 days	WBC (blood) Eosinophil (blood) Neutrophil (blood) Lymphocyte (blood) Monocyte (blood) EC50 OVA-response Contractility	<0.01 <0.001 <0.05 <0.01 NS <0.001 <0.001 <0.001
	Boskabady (2013) c	Guinea pig (both sexes, 6/6)	Carvacrol	160 µg/ml, orally	35 days	WBC (blood) Eosinophil (blood) Neutrophil (blood) Lymphocyte (blood) Monocyte (blood) EC50 OVA-response Contractility	<0.001 <0.001 <0.001 <0.001 <0.001 <0.001 <0.001 <0.001
10	Boskabady (2014) a	Guinea pig (both sexes, 6/6)	Carvacrol	40 µg/ml, orally	32 days	WBC (BALF) Eosinophil (BALF) Neutrophil (BALF) Lymphocyte (BALF) Monocyte (BALF)	<0.001 NS NS NS NS
	Boskabady (2014) b	Guinea pig (both sexes, 6/6)	Carvacrol	80 µg/ml, orally	32 days	WBC (BALF) Eosinophil (BALF) Neutrophil (BALF) Lymphocyte (BALF) Monocyte (BALF)	<0.001 <0.001 <0.05 <0.01 NS
	Boskabady (2014) c	Guinea pig (both sexes, 6/6)	Carvacrol	160 µg/ml, orally	32 days	WBC (BALF) Eosinophil (BALF) Neutrophil (BALF) Lymphocyte (BALF) Monocyte (BALF)	<0.001 <0.001 <0.01 <0.001 <0.001
11	Keyhanmanesh (2014)	Guinea pig (male, 15/15)	TQ	3 mg/kg, IP	32 days	WBC (BALF) Eosinophil (BALF) Neutrophil (BALF) Lymphocyte (BALF) Monocyte (BALF) EC50 OVA-response Contractility	NS <0.001 <0.001 <0.001 <0.001 <0.05 <0.01 NS
12	Pejman (2014)	Guinea pig (male, 15/15)	TQ	3 mg/kg, IP	32 days	WBC (BALF) Eosinophil (BALF) Neutrophil (BALF) Lymphocyte (BALF) Monocyte (BALF) EC50 OVA-response Contractility	NS <0.001 <0.001 <0.001 <0.001 <0.05 <0.01 NS
13	Zhou (2014)	BALB/c mice, (female, 8/8)	Thymol	4 mg/kg, IP	24 days	WBC (BALF) Eosinophil (BALF) Neutrophil (BALF) Macrophage (BALF) Lymphocytes (BALF)	<0.05 <0.01 <0.05 NS <0.05
	Zhou (2014)	BALB/c mice, (female, 8/8)	Thymol	8 mg/kg, IP	24 days	WBC (BALF) Eosinophil (BALF) Neutrophil (BALF) Macrophage (BALF) Lymphocytes (BALF)	<0.05 <0.01 <0.01 NS <0.01
	Zhou (2014)	BALB/c mice, (female, 8/8)	Thymol	16 mg/kg, IP	24 days	WBC (BALF) Eosinophil (BALF) Neutrophil (BALF) Macrophage (BALF) Lymphocytes (BALF)	<0.01 <0.01 <0.01 <0.5 <0.01
14	Saadat (2015) a	Guinea pig (male, 15/15)	TQ	3 mg/kg, IP	32 days	WBC (BALF) Eosinophil (BALF) Neutrophil (BALF) Lymphocytes (BALF) Monocytes (BALF) EC50 OVA-response Contractility	<0.01 <0.001 <0.001 <0.01 <0.05 NS <0.001 <0.05
	Saadat (2015) b	Guinea pig (male, 15/15)	α-hederin	0.3 mg/kg, IP	32 days	WBC (BALF) Eosinophil (BALF) Neutrophil (BALF) Lymphocytes (BALF) Monocytes (BALF) EC50 OVA-response Contractility	<0.001 <0.001 <0.01 <0.001 <0.001 <0.05 <0.001 <0.001
	Saadat (2015) c	Guinea pig (male, 15/15)	α-hederin	3 mg/kg, IP	32 days	WBC (BALF) Eosinophil (BALF) Neutrophil (BALF) Lymphocytes (BALF) Monocytes (BALF) EC50 OVA-response Contractility	<0.01 <0.001 <0.05 <0.001 <0.01 NS <0.001 <0.05
15	Khaldi (2018)	Rat (male, 6/6)	*N. sativa*	4 mL/kg/day, orally	27 days	WBC (BALF) Lymphocytes (BALF) Monocytes (BALF) Granulocyte (BALF)	<0.01 NS <0.05 <0.01
16	Rafique (2018) a	Guinea pig (Both sexes, 6/6)	*N. sativa*	5 mg/kg, orally	25 days	WBC (BALF) Eosinophil (BALF) Neutrophil (BALF) Lymphocytes (BALF) Monocytes (BALF)	<0.001 <0.001 NS NS NS
	Rafique (2018) b	Guinea pig (Both sexes, 6/6)	*N. sativa*	5 mg/kg, orally	25 days	WBC (blood) Eosinophil (blood) Neutrophil (blood) Lymphocytes (blood) Monocytes (blood)	NS <0.001 NS NS NS

BALF: bronchoalveolar lavage fluid, I.P: intra-peritoneal, NS: non-significant, OVA: ovalbumin, TQ: thymoquinone, WBC: white blood cell.

### Effectiveness

#### Effect of NS and its ingredients on total WBC count

**Figure 2:**
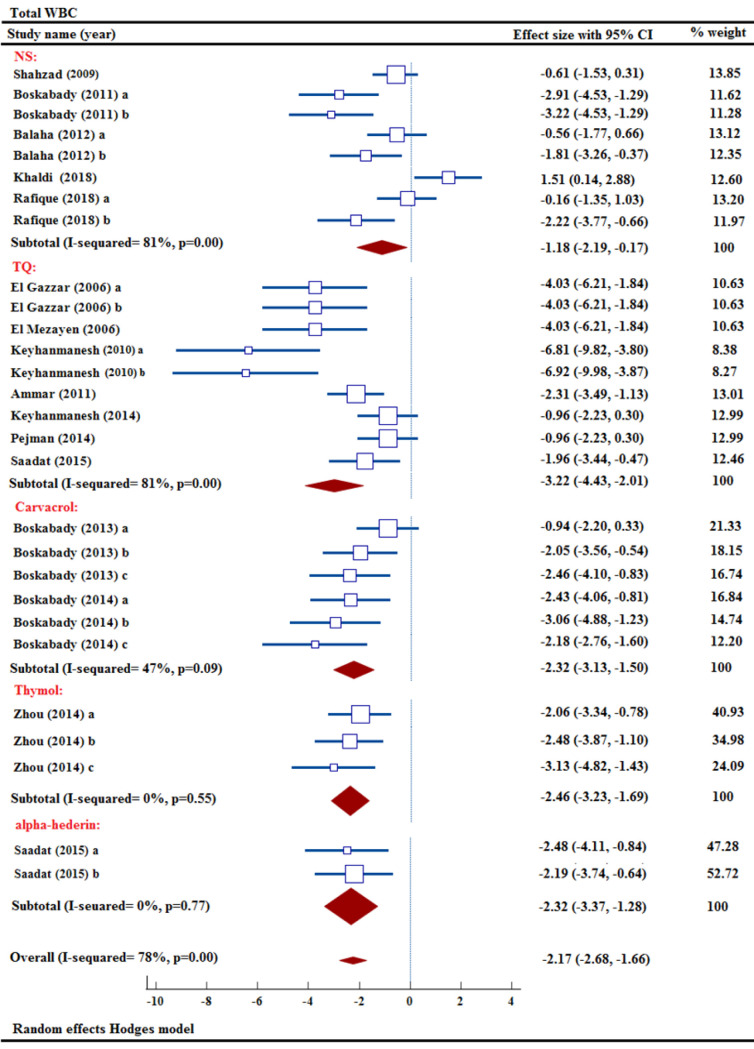
Forest plot detailing standardized mean differences (SMD) and 95% confidence intervals (CIs) in the studies reporting the effect of NS, TQ, carvacrol, thymol, and α-hederin on total WBC count in intervention groups compared to OVA-induced asthma group.

Among the sixteen studies analyzing total WBC count, five focused on various concentrations of NS extract and their impact on total WBC count (Balaha et al. 2012; Boskabady et al. 2011; Khaldi et al. 2018; Rafique et al. 2018; Shahzad et al. 2009). These five studies demonstrated that treatment with various levels of NS extract resulted in a marked reduction in total WBC count. The meta-analysis findings showed that NS extract effectively decreased the total WBC count in comparison to the asthma group [n= 112, SMD= -1.18, 95% CI (-2.19 to –0.17), p<0.05] (Figure 2).

The effects of carvacrol at various levels on total WBC count were examined in two studies, both studies showed markedly decreased total WBC (Boskabady and Jalali 2013; Boskabady et al. 2014). By analyzing data from two studies with various levels of carvacrol, the meta-analysis determined that the intervention group had reduced total WBC counts compared to the model group [n= 72, SMD= -2.32, 95% CI (-3.13 to -1.50), p<0.001] (Figure 2).

The effect of TQ treatment on total WBC count was studied in eight different research projects, revealing a significant decline in WBC levels (Ammar el et al. 2011; El Gazzar et al. 2006a; El Gazzar et al. 2006b; El Mezayen et al. 2006; Keyhanmanesh et al. 2010; Keyhanmanesh et al. 2014; Pejman et al. 2014; Saadat et al. 2015). The meta-analysis revealed a notable decrease in total WBC count in the TQ intervention group [n= 174, SMD= -3.22, 95% CI (-4.43 to -2.10), p<0.001] (Figure 2).

The effects of three different concentrations of thymol on total WBC were investigated (Zhou et al. 2014). According to the meta-analysis findings, thymol was shown to cause a notable reduction in the total WBC count [n= 48, SMD= -2.46, 95% CI (-3.23 to -1.69), p<0.001] (Figure 2).

The significant effects of α-hederin on total WBC count were evident in a study utilizing two different concentrations (Saadat et al. 2015). The meta-analysis findings indicated that α-hederin resulted in a notable reduction in the total WBC count [n= 60, SMD= -2.32, 95% CI (-3.37 to -1.28), p<0.001] (Figure 2).

The overall meta-analysis demonstrated that NS extract and its active ingredients successfully decreased total WBC count [n= 466, SMD= -2.17, 95% CI (-2.68 to -1.66), p<0.001] (Figure 2).

### Effect of NS and its ingredients on eosinophil count 

**Figure 3:**
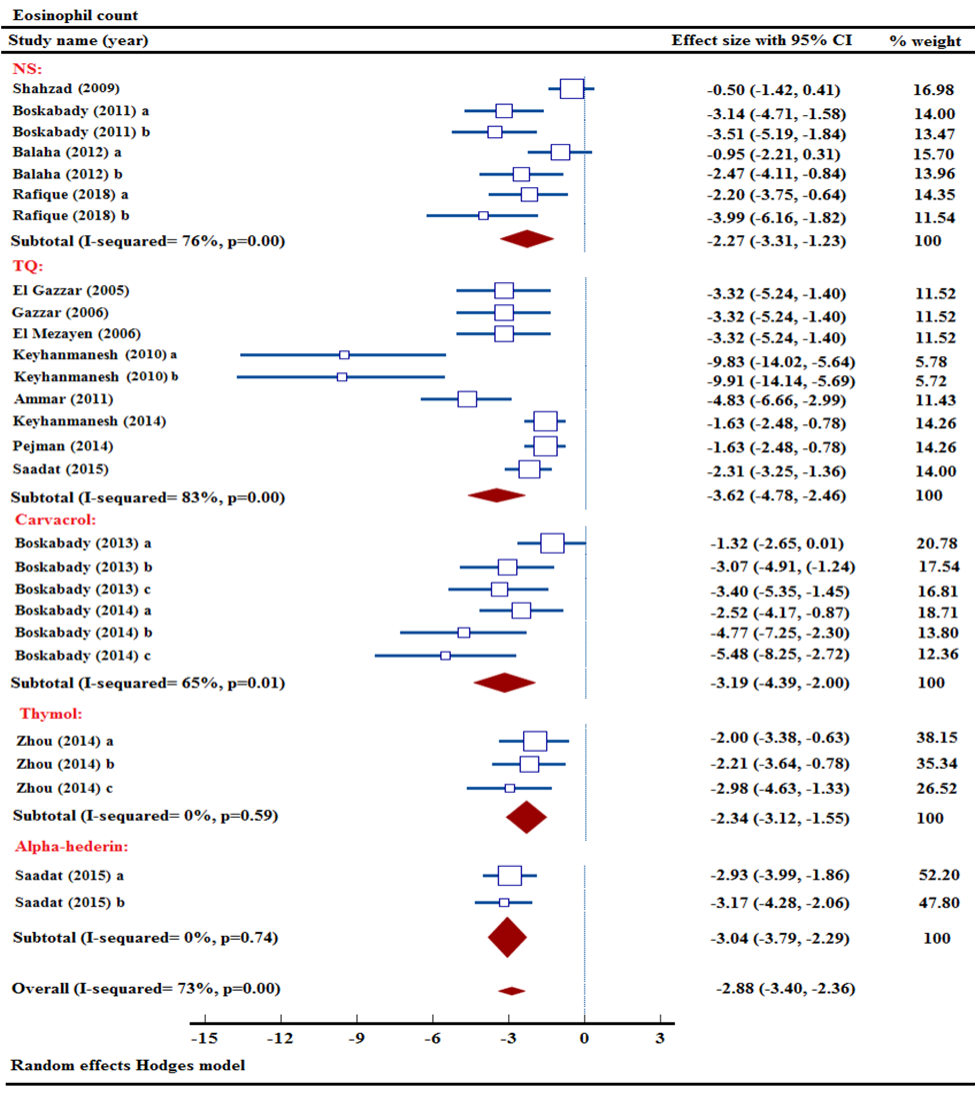
Forest plot detailing standardized mean differences (SMD) and 95% confidence intervals (CIs) in the studies reporting the effect of NS, TQ, carvacrol, thymol, and α-hederin on eosinophil count in intervention groups compared to OVA-induced asthma group.

Out of the sixteen studies assessing eosinophil count, four studies focused on the impact of NS extract treatment on eosinophil levels (Balaha et al. 2012; Boskabady et al. 2011; Rafique et al. 2018; Shahzad et al. 2009). Through a meta-analysis incorporating data from 4 studies, it was found that NS extract had a significant effect in reducing of eosinophil count [n= 100, SMD= -2.27, 95% CI (-3.31 to -1.23), p<0.001] (Figure 3).

The impact of treatment with three distinct levels of carvacrol on eosinophil counts was studied in two research trials (Boskabady and Jalali 2013; Boskabady et al. 2014). Two studies pooled data indicated a significant decrease in eosinophil levels as a result of carvacrols interventions [n= 72, SMD= -3.19, 95% CI (-4.39 to -2.00), p<0.001] (Figure 3).

Eight studies have established the impactful effectiveness of TQ on lowering eosinophil numbers across multiple dosage levels (Ammar el et al. 2011; El Gazzar et al. 2006a; El Gazzar et al. 2006b; El Mezayen et al. 2006; Keyhanmanesh et al. 2010; Keyhanmanesh et al. 2014; Pejman et al. 2014; Saadat et al. 2015). After meta-analyzing data from 8 different studies, it was found that TQ had a noteworthy impact on lowering eosinophil levels [n= 174, SMD= -3.62, 95% CI (-4.78 to -2.46), p<0.001] (Figure 3).

The use of three different doses of thymol in a research experiment showed that thymol prevented increased eosinophil level in an animal model of asthma (Zhou et al. 2014). The meta-analysis of combined data showed that thymol effectively decreased eosinophil count [n= 48, SMD= -2.34, 95% CI (-3.12 to -1.55), p<0.001] (Figure 3).

The study on eosinophil numbers revealed the reducing effects of two concentrations of α-hederin intervention (Saadat et al. 2015). The combination of data in a meta-analysis illustrated the significant impact of α-hederin on lowering eosinophil level in an experimental asthma model [n= 60, SMD= -3.04, 95% CI (-3.79 to -2.29), p<0.001] (Figure 3).

In the comprehensive meta-analysis, treatment with NS, TQ, carvacrol, thymol, and α-hederin resulted in a significant decrease in eosinophil numbers compared to the OVA group [n= 454, SMD= -2.88, 95% CI (-3.40 to -2.36), p<0.001] (Figure 3).

### Effect of NS and its ingredients on lymphocyte count

**Figure 4:**
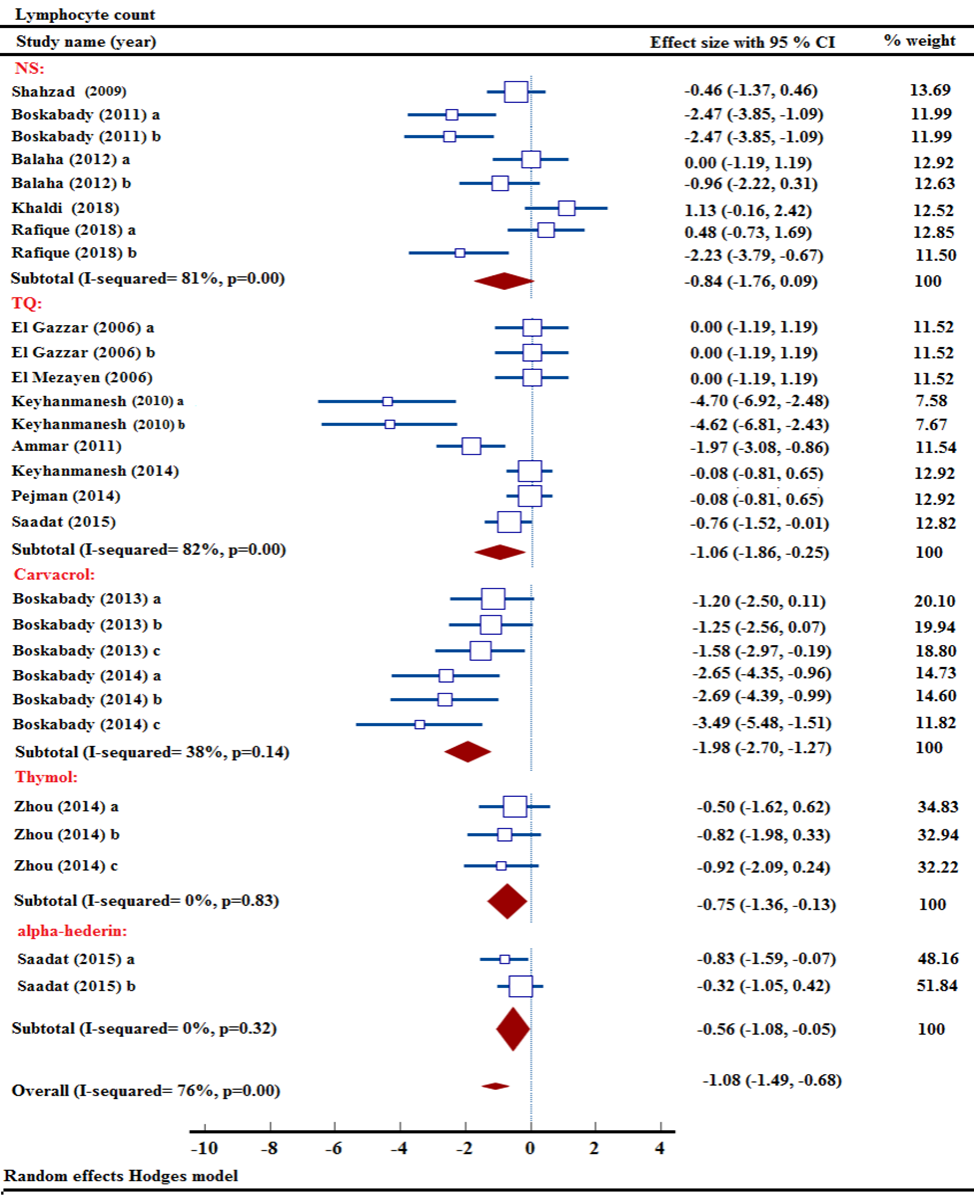
Forest plot detailing standardized mean differences (SMD) and 95% confidence intervals (CIs) in the studies reporting the effect of NS, TQ, carvacrol, thymol, and α-hederin on lymphocyte count in intervention groups compared to OVA-induced asthma group.

Five studies from a pool of sixteen explored research, on the effects of NS, investigated the lymphocyte count (Balaha et al. 2012; Boskabady et al. 2011; Khaldi et al. 2018; Rafique et al. 2018; Shahzad et al. 2009). After combining data from 5 studies, a meta-analysis concluded that NS extract did not lower lymphocyte levels [n= 112, SMD= -0.84, 95% CI (-1.76 to 0.09)] (Figure 4).

The effects of carvacrol on lymphocyte count were investigated in two studies using it at three different concentrations (Boskabady and Jalali 2013; Boskabady et al. 2014). Carvacrol was found to significantly reduce lymphocyte level in a meta-analysis combining data from 2 studies [n= 72, SMD= -1.98, 95% CI (-2.70 to -1.27), p<0.001] (Figure 4).

The impact of TQ on lymphocyte numbers has been assessed in eight distinct studies with various concentrations (Ammar el et al. 2011; El Gazzar et al. 2006a; El Gazzar et al. 2006b; El Mezayen et al. 2006; Keyhanmanesh et al. 2010; Keyhanmanesh et al. 2014; Pejman et al. 2014; Saadat et al. 2015). The synthesis of data from 8 studies revealed a substantial influence of TQ in lowering lymphocyte level in OVA-induced animal model of asthma [n= 174, SMD= -1.06, 95% CI (-1.86 to -0.25), p<0.05] (Figure 4).

The effects of thymol on lymphocyte count were measured at different concentrations in a study involving an animal model of OVA-induced asthma (Zhou et al. 2014). Thymol was found to have a substantial impact on decreasing lymphocyte level in a meta-analysis of combined data [n= 48, SMD= -0.75, 95% CI (-1.36 to -0.13), p<0.05] (Figure 4).

Research explored the effects of two distinct concentrations of α-hederin on lymphocyte counts in asthmatic animals (Saadat et al. 2015). A comprehensive analysis of combined information revealed that α-hederin had a noteworthy impact on lowering lymphocyte levels [n= 60, SMD= -0.56, 95% CI (-1.08 to -0.05), p<0.05] (Figure 4).

The overall meta-analysis findings showed that NS, TQ, carvacrol, thymol, and α-hederin resulted in a significant decrease in lymphocyte count when compared to the non-treated asthma group [n= 466, SMD= -1.08, 95% CI (-1.49 to -0.68), p<0.001] (Figure 4).

### Effect of NS and its ingredients on monocyte count

**Figure 5:**
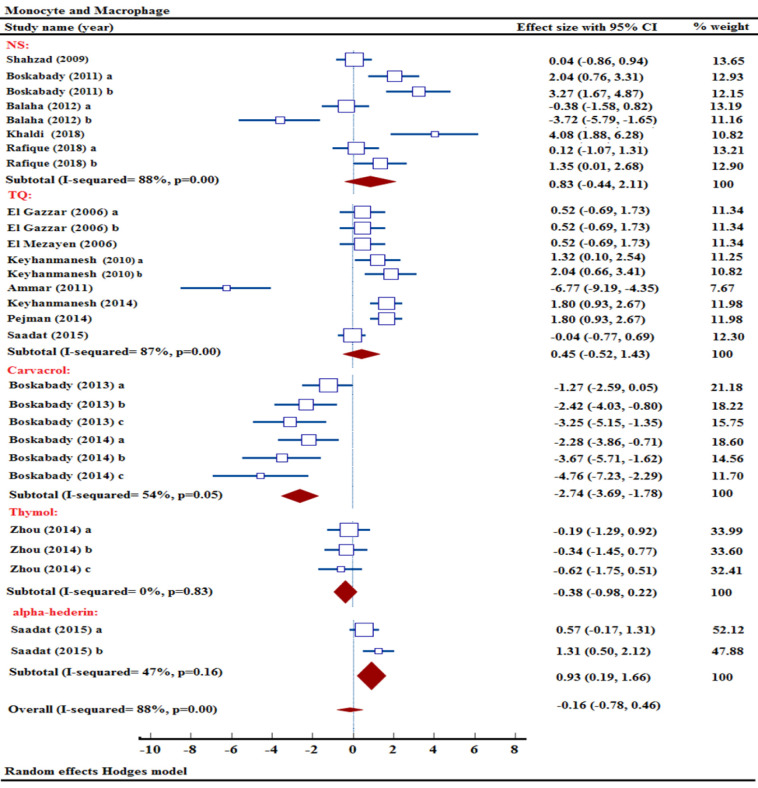
Forest plot detailing standardized mean differences (SMD) and 95% confidence intervals (CIs) in the studies reporting the effect of NS, TQ, carvacrol, thymol, and α-hederin on monocyte and macrophage count in intervention groups compared to OVA-induced asthma group.

Five out of sixteen studies have examined the impact of NS treatment on monocyte levels (Balaha et al. 2012; Boskabady et al. 2011; Khaldi et al. 2018; Rafique et al. 2018; Shahzad et al. 2009). The results of a meta-analysis pooling data from 5 studies demonstrated that NS extract was not effective in reducing monocyte levels [n= 112, SMD= 0.83, 95% CI (-0.44 to 2.11)] (Figure 5).

Two studies examined the impact of three distinct concentrations of carvacrol on monocyte counts in OVA-sensitized animals (Boskabady and Jalali 2013; Boskabady et al. 2014). Combining data from 2 studies, a meta-analysis found that carvacrol has a substantial impact on decreasing monocyte levels [n= 72, SMD= -2.74, 95% CI (-3.69 to -1.78), p<0.001] (Figure 5).

The influence of various TQ levels on monocyte quantity was analyzed in eight different research studies (Ammar el et al. 2011; El Gazzar et al. 2006a; El Gazzar et al. 2006b; El Mezayen et al. 2006; Keyhanmanesh et al. 2010; Keyhanmanesh et al. 2014; Pejman et al. 2014; Saadat et al. 2015). An examination of combined data from 8 studies revealed that TQ did not impact lymphocyte levels [n= 174, SMD= 0.45, 95% CI (-0.52 to 1.43)] (Figure 5).

The potential impact of different thymol concentrations on monocyte levels in a study has been explored (Zhou et al. 2014). Thymol was found to have no significant impact on reducing monocyte levels in a meta-analysis of combined data [n= 48, SMD= -0.38, 95% CI (-0.98 to 0.22)] (Figure 5).

The impact of two concentrations of α-hederin on monocyte count was analyzed in an experimental model of asthma (Saadat et al. 2015). Pooled data analysis demonstrated that the α-hederin had a notable impact on lowering monocyte counts [n= 60, SMD= 0.93, 95% CI (0.19 to 1.66), p<0.05] (Figure 5).

The comprehensive meta-analysis showed that the NS extract and its active components did not decrease the monocyte count in comparison to the untreated asthma group [n= 466, SMD= -0.16, 95% CI (-0.78 to 0.46)] (Figure 5).

### Effect of NS and its ingredients on neutrophil count

**Figure 6:**
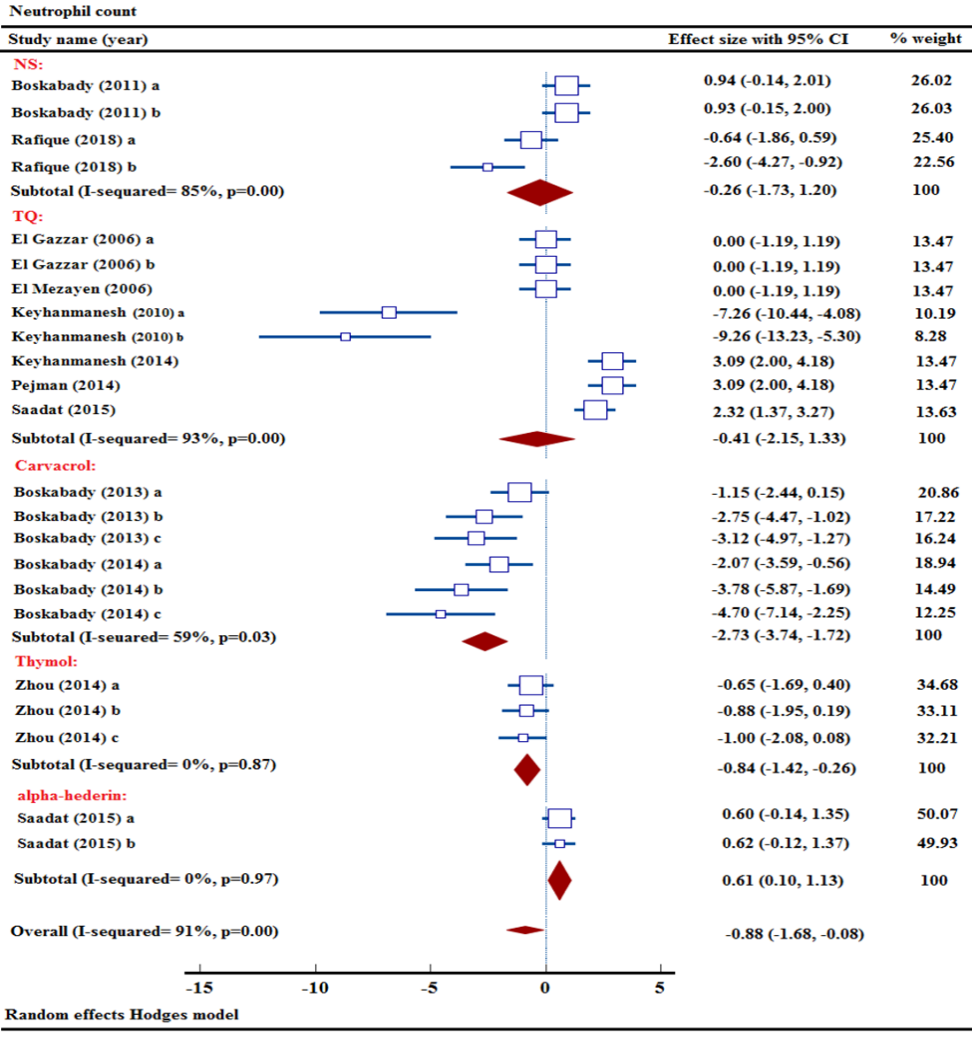
Forest plot detailing standardized mean differences (SMD) and 95% confidence intervals (CIs) in the studies reporting the effect of NS, TQ, carvacrol, thymol, and α-hederin on neutrophil count in intervention groups compared to OVA-induced asthma group.

Out of the thirteen research projects examining neutrophil levels, two were specifically focused on studying the impact of NS extract (Boskabady et al. 2011; Rafique et al. 2018). Combining data from two studies using various concentrations of NS extract, a meta-analysis found no significant impact on neutrophil counts [n= 56, SMD= -0.26, 95% CI (-1.73 to 1.20)] (Figure 6).

Furthermore, the effects of carvacrol on neutrophil count were studied in two research trials utilizing its three different concentrations (Boskabady and Jalali 2013; Boskabady et al. 2014). Combining data from 2 studies, a meta-analysis revealed a notable decrease in neutrophil levels due to carvacrol treatment [n= 72, SMD= -2.73, 95% CI (-3.74 to -1.72), p<0.001] (Figure 6).

Seven studies were carried out to study the influence of TQ concentrations on neutrophil count (El Gazzar et al. 2006a; El Gazzar et al. 2006b; El Mezayen et al. 2006; Keyhanmanesh et al. 2010; Keyhanmanesh et al. 2014; Pejman et al. 2014; Saadat et al. 2015). Combining data from 7 studies examining various concentrations of TQ, a meta-analysis found no notable impact on neutrophil counts [n= 154, SMD= -0.41, 95% CI (-2.15 to 1.33)] (Figure 6).

A study revealed the effects of three different concentrations of thymol on neutrophil count in an animal model of asthma (Zhou et al. 2014). Combining data from multiple sources, a meta-analysis concluded that thymol did not have a notable impact on lowering neutrophil level [n= 48, SMD= -0.84, 95% CI (-1.42 to -0.26), p<0.01] (Figure 6).

Neutrophil numbers in response to α-hederin level were documented in a research investigation (Saadat et al. 2015). A significant reduction in neutrophil level was observed in a meta-analysis of pooled data, indicating the effectiveness of α-hederin [n= 60, SMD= 0.61, 95% CI (0.10 to 1.13), p<0.05] (Figure 6).

In a comprehensive meta-analysis, NS, thymol, carvacrol, α-hederin, and TQ were found that carvacrol, thymol, and α-hederin significantly decrease neutrophil counts across thirteen studies [n= 390, SMD= -0.88, 95% CI (-1.68 to -0.08), p<0.05] (Figure 6).

### Effect of NS and its ingredients on EC50 and maximum response value

**Figure 7:**
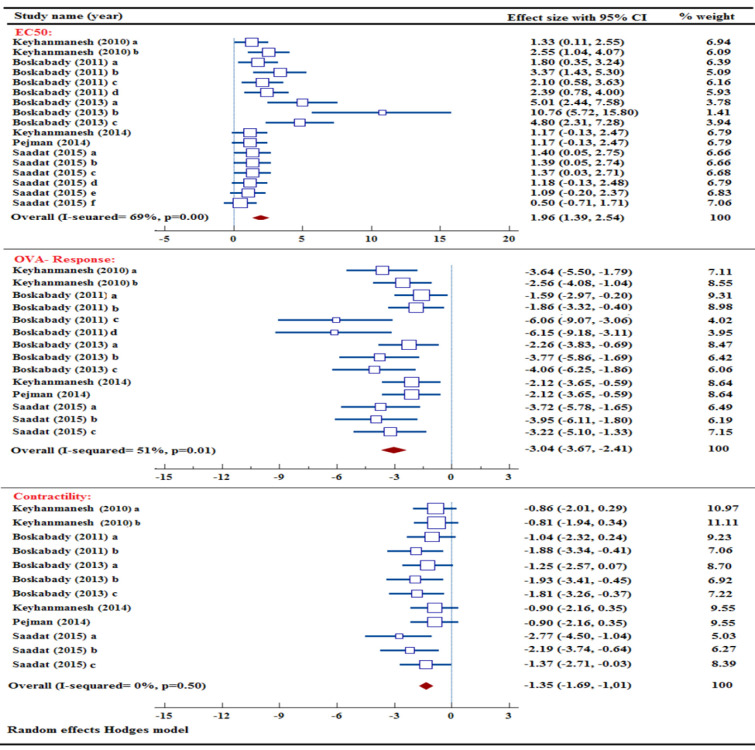
Forest plot detailing standardized mean differences (SMD) and 95% confidence intervals (CIs) in the studies reporting the effect of NS and its active ingredients on EC50, OVA-response, and contractility values in intervention groups compared to OVA-induced asthma group. EC50: half maximal effective concentration.

As an outcome measure, the EC_50_ value was employed in six research studies in OVA-induced animal model of asthma (Boskabady and Jalali 2013; Boskabady et al. 2011; Keyhanmanesh et al. 2010; Keyhanmanesh et al. 2014; Pejman et al. 2014; Saadat et al. 2015). The study results indicated that NS extract, TQ, α-hederin, and carvacrol were able to raise the EC_50_ level in comparison to the untreated asthma model group. A combination of six studies showed a significant effect of NS extract, TQ, α-hederin, and carvacrol in increasing EC_50_ value [n=208, SMD= 1.96, 95% CI (1.39 to 2.54), p<0.001] (Figure 7).

OVA-response was utilized as a measure of outcome in six different studies in animal models of asthma (Boskabady and Jalali 2013; Boskabady et al. 2011; Keyhanmanesh et al. 2010; Keyhanmanesh et al. 2014; Pejman et al. 2014; Saadat et al. 2015). In comparison to the untreated asthma model group, the results indicated a decrease in OVA response with NS extract, TQ, α-hederin, and carvacrol. A meta-analysis of seven studies demonstrated a noteworthy influence of NS extract, TQ, α-hederin, and carvacrol in decreasing the OVA response [n=172, SMD= -3.04, 95% CI (-3.67 to -2.41), p<0.001] (Figure 7).

Six different studies utilized tracheal contractility as a measure in OVA-sensitized animals (Boskabady and Jalali 2013; Boskabady et al. 2011; Keyhanmanesh et al. 2010; Keyhanmanesh et al. 2014; Pejman et al. 2014; Saadat et al. 2015). The results revealed that NS extract, TQ, α-hederin, and carvacrol were effective in decreasing tracheal contractility when compared to the non-treated asthma model group. Six studies revealed a marked reduction in tracheal contractility as a result of NS extract, TQ, α-hederin, and carvacrol [n=148, SMD= -1.35, 95% CI (-1.69 to -1.01), p<0.001] (Figure 7).

## Discussion

The key findings of the current preclinical systematic review on the effectiveness of NS and its compounds on the OVA-induced asthma in animal studies were: 

1- Reduction in the total WBC, eosinophils, lymphocytes, and neutrophils count, 

2- The use of NS and its components led to improved respiratory function by reducing airway responsiveness to contractile agents such as methacholine and OVA, through improving EC_50_, and maximal airway response to contractile agents and airway responsiveness to ovalbumin.

Asthma has been categorized into different phenotypes including clinical, induced, inflammatory, and allergic (Kuruvilla et al. 2019). Inflammation is a critical factor in the development of asthma. Asthma-related airway inflammation prompts the activation of different mediators and immune cells (Aslani et al. 2020; Aslani et al. 2016b). Obvious clinical manifestations in asthma, such as cough and shortness of breath, have been revealed as a result of bronchial inflammation and airway obstruction (Hammad and Lambrecht 2021). Lymphokines, growth factors, inhibitory cytokines, chemokines, and proinflammatory cytokines are all examples of the types of cytokines associated with asthma pathogenesis (Hammad and Lambrecht 2021). Research conducted on both humans and animals has revealed a rise in total WBCs and differential cells within the airways of individuals with asthma (Peebles and Aronica 2019). In the OVA-induced asthma model, inflammatory cell infiltration has been observed in both the blood and broncho-alveolar lavage (BAL) fluid samples (Ahmadi et al. 2016; Aslani et al. 2022b). 

In the included studies, high levels of eosinophils were observed in BAL fluid. The use of NS and its active components showed improvements in eosinophil levels. The current meta-analysis showed that NS had a significant impact on decreasing eosinophil levels. Moreover, subgroup analysis revealed that the NS extract, along with its active compounds including TQ, carvacrol, thymol, and alpha-hederin, demonstrated a decrease in BAL fluid eosinophil levels. On the other hand, the results of the studies revealed that the level of lymphocytes was higher in the BAL fluid in asthma groups. While certain studies have shown that NS extract intervention can lower lymphocyte levels, others have found conflicting results. The current meta-analysis further illustrated that the overall impact of NS extract on lowering lymphocyte levels was not deemed significant. An interesting outcome emerged from the subgroup analysis of NS active ingredients: TQ, carvacrol, thymol, and alpha-hederin were all shown to significantly reduce lymphocyte levels, a result that was confirmed by the current meta-analysis. Further investigation may reveal that NS active ingredients have a greater impact on reducing lymphocyte numbers compared to NS extract. The current meta-analysis did not find any significant correlations between NS and its active ingredients with monocyte and macrophage counts. Surprisingly, carvacrol had a significant impact on decreasing monocyte and macrophage counts, while NS extract, thymol, TQ, and alpha-hederin did not show significant effects. Because the studies included a small sample size, it was not feasible to conduct subgroup analysis on the dosage of NS extract and its active ingredients like thymol, TQ, and alpha-hederin, potentially impacting the findings of this study. On the other hand, it is possible that under the influence of NS and its active ingredients, the phenotype of immune cells has undergone changes in asthma condition, necessitating more in-depth studies. Despite the noticeable decrease in neutrophil levels seen with NS extract and its active ingredients in the current meta-analysis, conflicting reports were found in various studies. In the current meta-analysis, there were no significant changes in neutrophil counts following intervention with NS or TQ extract. Following the alpha-hederin intervention, a notable rise in neutrophil levels was observed in the meta-analysis results. Conversely, there was a marked reduction in neutrophil numbers as a result of the intervention utilizing carvacrol and thymol. Additional research is needed to investigate the impact of NS and its active components on neutrophil levels, given that the limited sample sizes in certain studies could have influenced the outcomes of the meta-analysis.

The presence of eosinophils has been identified as a significant indicator for predicting asthma exacerbations (Bjerregaard et al. 2017). Studies have demonstrated that eosinophils are responsible for triggering the release of a range of mediators including radical oxygen species, major basic proteins, and cytokines (Lombardi et al. 2022). Eosinophils release granular products, causing damage to epithelial cells and worsening asthma attacks. In addition, the release of transforming growth factor (TGF)-β by eosinophils has been discovered to have a significant impact on airway remodeling and fibrosis (Nakagome and Nagata 2018). On the other hand, the function of monocytes and macrophages in asthma patients has been clearly established. Patients with fatal asthma display higher levels of monocytes in their lower airway mucosa and alveoli (Watanabe et al. 2023). Macrophages contribute to eosinophilic airway inflammation by releasing key cytokines like IL-6, tumor necrosis factor alpha (TNF-α), and IL-8, as well as generating reactive oxygen species to facilitate the production of IL-5 from CD4+ T cells (Zhu et al. 2020). In the airways of asthmatic patients, macrophages have been discovered to amplify the neutrophilic immune response through the secretion of IL-17, particularly in severe cases (Hynes and Hinks 2020). Recent findings from studies on animal models of asthma induced by OVA combined with obesity indicate a significant impact of IL-17 immune response on worsening asthma symptoms (Aslani et al. 2022c).

The immune system-modifying capabilities of NS and its active ingredients have been revealed in multiple research studies. Findings indicate that NS and active elements exhibit characteristics such as immune stimulant or immunosuppressant properties (Wei et al. 2022). Furthermore, NS has been shown to possess antioxidant and radical scavenging properties that have been linked to the enhancement of respiratory conditions (Saadat et al. 2021). Research on OVA-induced asthma in animals reveals that the hydroethanolic extract of NS, black seed oil, and NS fixed oil can effectively reduce total WBC, eosinophils, lymphocytes, and macrophages in BAL fluid (Saadat et al. 2021). Furthermore, the preventive properties of NS active compounds like TQ, carvacrol, thymol, and alpha-hederin have been demonstrated in limiting the rise of WBCs, lymphocytes, neutrophils, monocytes, and notably eosinophils in an animal model of OVA-induced asthma (Saadat et al. 2021).

An analysis of current data has revealed that NS and its active elements led to a prominent decrease in the total number of WBC, eosinophils, neutrophils, and lymphocytes. It is worthy to mention that the impact of NS and its active components on monocyte count did not show significance, suggesting a potential shift in the asthma phenotype from Th-2 to Th-1 that requires further investigation.

A notable feature of asthma is its association with airway hyperresponsiveness (AHR). Studies on animals utilize criteria such as EC_50_ and measure the increased airway responsiveness to constrictors like methacholine, histamine, and OVA-response in order to explore AHR (Boskabady et al. 2011; Pejman et al. 2014). In experiments involving OVA-induced asthma in animals, it was discovered that the EC_50_ values decreased significantly in the asthma model, suggesting a heightened sensitivity to constrictors (Boskabady et al. 2011). NS and its active ingredients have led to a significant shift in the concentration-response curve to contractile agents towards the right, resulting in a marked increase in EC_50_ and thereby improving airway responsiveness (Boskabady et al. 2011). A recent meta-analysis revealed that NS and its active components positively impacted lung function in animal research. In addition, the animal model of asthma exhibits a greater sensitivity of the airways to methacholine, histamine, and OVA, yet treatment with NS and other effective agents’ results in relaxation rather than contraction. The findings from the current meta-analysis suggest that NS and its active components showed preventative effects on AHR. A variety of mechanisms have been revealed regarding the therapeutic impact of NS and, specifically, TQ on airway relaxation.

One of the limitations of the present study is the lack of analysis of different concentrations of NS and its active ingredients. In addition, the method of using NS and its active ingredients was different (oral or I.P.). Conversely, while the sensitization and OVA challenge were employed for data analysis in accordance with the study’s criteria, differing durations of asthma induction prove to be a limitation. Finally, the lack of consistency and variability observed in both study design and experiment execution were identified as limitations in this study.

A current systematic review unveiled the potential protective properties of NS and its active compounds like TQ, carvacrol, α-hederin, and thymol in lowering airway inflammation and enhancing lung function in animal models of OVA-induced asthma.

## Data Availability

All data generated or analyzed during this study are included in this published article [and its supplementary information files].
